# The Assessment of Toxic Metals in Plants Used in Cosmetics and Cosmetology

**DOI:** 10.3390/ijerph14101280

**Published:** 2017-10-24

**Authors:** Agnieszka Fischer, Barbara Brodziak-Dopierała, Krzysztof Loska, Jerzy Stojko

**Affiliations:** 1 Department of Toxicology and Bioanalysis, Medical University of Silesia, 41-200 Sosnowiec, Poland; afischer@sum.edu.pl (A.F.); jstojko@sum.edu.pl (J.S.); 2 Institute of Water and Wastewater Engineering, Silesian University of Technology, 44-100 Gliwice, Poland; krzysztof.loska@polsl.pl

**Keywords:** plants, AAS, cadmium, lead, mercury

## Abstract

Heavy metals polluting the natural environment are absorbed by plants. The use of herbs as components of cosmetics may pose a health risk for humans. The aim of the study was to determine the concentrations of Pb, Cd and Hg in selected species of herbs (horsetail *Equisetum arvense*, nettle *Urtica dioica*, St. John’s wort *Hypericum perforatum*, wormwood *Artemisia absinthium*, yarrow *Achillea millefolium*, cottonwood *Solidago virgaurea*) self-collected from the natural environment in two different locations, and purchased in stores on the territory of Poland. The concentration of the metals studied was: 4.67–23.8 mg/kg Pb, 0.01–1.51 mg/kg Cd, 0.005–0.028 mg/kg Hg. Different concentrations of metals, depending on species and origin of plants, were found. The mean concentration of all studied metals was the lowest in St. John’s wort, and the highest in nettle. In herbs purchased in Polish stores, the concentration of Pb was higher than in plants self-collected in the natural environment.

## 1. Introduction

Human activity has greatly affected the biochemical and geological balance of many elements in the natural environment. Heavy metals are substantial environmental pollution because they can remain therein relatively long, are involved in the food chain and are subject to retention [1]. The content of heavy metals in the environment is conditioned by a number of factors, both natural (mineral composition of soil, climate), and anthropogenic (industry, agriculture, transport) [2]. Assessment of the degree of heavy metal pollution is particularly important for agriculture and crops used for other purposes, such as in phytotherapy or cosmetology [2].

The increasing demand for cosmetics and versatility of their use result in gradual development of the cosmetic industry. Over the last 20 years, global production of cosmetics has increased by an average of 4.5% a year [3,4]. In response to the increase in popularity of cosmetic products, an increased incidence of adverse reactions has been observed as well. However, users’ knowledge of cosmetic prescriptions and health safety of cosmetics used is growing.

Substances that can be found in cosmetic products and may be potentially dangerous include preservatives, fragrances, dyes, and metals [5]. Toxicity of heavy metals to the human body is well documented [6]. Even in low concentrations, some metals can show negative health effects that can damage internal organs and impair their functioning [3,7].

It is believed that the pollution with heavy metals that can be found in cosmetic products is unavoidable due to their ubiquity. However, they should be removed where it is technically possible [8]. It is suggested that cosmetics should not contain elements such as arsenic, cadmium, lead, mercury, beryllium, selenium [9]. However, some metals, such as cadmium and lead, can be released by metal components used in technological processes of cosmetic products [10].

The most commonly detected heavy metals in cosmetics are lead, cadmium, mercury, chromium, nickel and copper [11,12]. These metals can be found in various cosmetic products such as shampoos, creams and cosmetics used for make-up. Prescription ingredients used and improper treatment of raw materials affect the presence of heavy metal contaminants in cosmetics [3,13]. The type of ingredient used, the type of dye used in cosmetics, and improper purification of the raw material affect the presence of heavy metal contaminants in cosmetics.

Contaminated cosmetic products can cause adverse health effects [3,10,14,15,16,17,18,19]. Metals found in cosmetics can act locally on the skin or accumulate in the body after absorption and cause systemic toxic effects [6]. Literature data indicates that toxic metals may be present in commercially available cosmetics in amounts that pose a risk for human health [19,20,21].

At present, the popularity of natural cosmetics is increasing [22,23]. These products are considered potentially safer for health than synthetic raw materials. Many people prepare cosmetics with the use of vegetable ingredients, including self-harvested from the natural environment. Literature data indicates that cosmetic plants can be severely polluted with heavy metals. Information on this issue avoids or minimizes the use of plants contaminated with toxic metals in the production process [24].

Among plants found in Poland, the most commonly used in cosmetology are horsetail and nettle. These plants are used in the care of skin, hair and nails. Horsetail is characterized by a high concentration of minerals, especially silicon. These ingredients restore skin vitality and improve its appearance, with stimulating and revitalizing effects. Nettle extracts are commonly used as hair strengthening agents, preventing dandruff and seborrhea in the skin. St. John’s Wort stimulates the regeneration process in the skin, counteracts wrinkles and promotes epidermis renewal, as well as preventing skin hyperemia, making it useful in the care of sensitive or vascular skin types, or even skin prone to rosacea. Wormwood is used as a supplement to baths with a strengthening effect, improving skin circulation. Yarrow and cottonwood have antiseptic, anti-inflammatory and astringent effects, they are used to deal with seborrhoea and acne. In ready-made cosmetic products the plants are most often found in the form of extracts or essential oils in the concentration of 0.5–10% [25,26].

The aim of the study was to determine the concentrations of toxic metals (lead, cadmium, and mercury) in plant species commonly found in the natural environment in Poland (nettle, yarrow, St. John’s wort, wormwood, horsetail, cottonwood), having a wide range of cosmetic applications. The concentration of Cd, Pb and Hg in wild plants in two different locations and in commercial plant products was examined. A comparative analysis of the occurrence of 3 metals, depending on origin and location of plant collection, was made. The results of the research show the degree of pollution with heavy metals in the plant materials tested, obtained independently from the natural environment and purchased commercially in a readymade form.

## 2. Materials and Methods

The raw materials from the following species of plants were studied: nettle (*Urtica dioica* L.), yarrow (*Achillea millefolium* L.), St. John’s wort (*Hypericum perforatum* L.), wormwood (*Artemisia absinthium* L.), horsetail *(Equisetum arvense* L.), cottonwood (*Solidago virgaurea* L.). The selected plant species have been widely used as herbal ingredients in medicine, and are in cosmetology as well [12,22,23]. They are among species commonly found in Poland, and popular among the local population. Vegetable materials that were used include nettle leaf (*Urticae folium*), yarrow herb (*Millefolia herba*), St. John’s wort herb (*Hyperici herba*), wormwood herb (*Absinthii herba*), horsetail herb (*Equiseti herba*), cottonwood herb (*Solidaginis herba*). The identification of the plants was made by Department of Pharmaceutical Botany and Herbal Medicine Medical University of Silesia.

**Figure 1:**
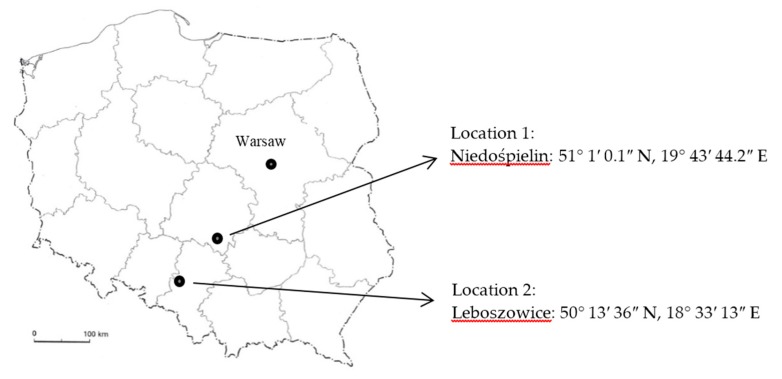
Plant collection area, Poland.

The research tested 3 separate groups of plants. Two of them were plants growing in natural areas. These were the wild herbs collected in Poland. The areas of collection were randomly selected—located in the southern part of the country, approximately 160 km apart. The researched locations are location 1—Niedośpielin (Łódzkie Province) and location 2—Leboszowice (Silesian Province)—Figure 1.

The area studied is characterized by a high coefficient of mercury enrichment in the soil. This is due to the large urbanization of the area and heavy pollution from many different industrial plants (steelworks, mines, CHPs—combinated heat and power) [27].

The selected plant collection locations represented similar natural habitats. In both cases, these were plant communities in the form of meadows or wastelands surrounding small rural villages. In each case, the locations of collection were 100 m away (minimum) from roads and fields to reduce the presence of local pollutants from these sources.

Due to the fact that car traffic is a major source of pollution in cities, regulations governing emissions from this source have been introduced. In Poland, the Directive of the European Parliament was adopted, which in 1987 determined the permitted lead content in petrol at 0.15 to 0.40 g/dm^3^. In Poland, standards for gasoline that reduce lead content similar to Western European standards have been in force since 1992 [28].

The plant collection process and the preparation of plants were to reproduce the process by which plant materials are harvested for individual use. For this purpose, popular information according to which the plant collection period for phytoterapeutic purposes is summer months (June–September) was used.

The plants studied are perennial species whose vegetation period in Poland falls on the months between May and September. Raw materials were fully grown during the growing season. The average height of plants collected for the research was about 50–60 cm.

From natural areas, herbs (horsetail, St. John’s wort, wormwood, yarrow, cottonwood) and nettle leaves were used for the study.

After collection, raw materials were dried in a shady, dry and airy place until dry mass was obtained. At least 10 randomly selected samples of plants were crushed in a porcelain mortar, and after standardization on sieves (1 mm in diameter) an average sample for the research was prepared. Samples of six species of wild plants growing in two distinct locations were taken for the research, *n* = 9—Figure 1.

A separate group of plant samples came from herbal products purchased in Polish stores. The herbal products purchased were identical to the self-collected raw materials. According to producers’ information, herbal raw materials came from Poland (contracted crops and the purchase of plants from the so-called natural state).

Two separate commercial products of each plant species were purchased for the research from different manufacturers (*n* = 12). Both self-collected and purchased raw materials were in a dried form. Approximately 20 g of dry vegetable material was used to make an average sample to be representative of the commercial product. The average sample was crushed in a porcelain mortar. Samples were stored in plastics.

### 2.1. Testing the Concentration of Pb and Cd in Plant Samples

Approximately 0.4 g of raw vegetable material was weighed from each average sample. Samples were subject to wet mineralization (spectrally pure nitric acid, Merck, Germany), (MAGNUM II microwave digester—ERTEC-Poland). The samples were placed in a Teflon vessel one by one and mineralization was added. Mineralization was a two-stage procedure. The first stage lasted 2 min at 20 bar of maximum pressure and 255 °C of maximum temperature, whereas the second stage was 6 min at 45 bar of maximum pressure and 285 °C of maximum temperature. The post-mineralization solution was transferred to a 25 cm^3^ flask and then diluted to the ml mark with re-distilled water. The concentration of Cd and Pb was marked using the flameless AAS method (SpectrAA 880Z-VARIAN).

### 2.2. Testing Hg Concentration in Plant Samples

Each average sample was used to weigh 50 mg of plant material for testing. Measurement of Hg concentration was made using atomic absorption spectroscopy with the AMA 254 (Advanced Mercury Analyzer, Altec Ltd., Prague, Czech Republic) analyser. The measurement procedure applied allows for the release of mercury from all chemical forms of this element (organic and inorganic bonds), and transformation of mercury to the atomic form. The final result of the marking indicates the total Hg concentration in the sample being analysed. For each plant sample, 2 to 3 repetitions of markings were made to obtain reproducibility of the results. The limit of detection for this method is 0.01 ng, 0.01 µg/g of mercury in the sample.

### 2.3. Verification of Marking Correctness

The correctness of the methodology used was verified by the reference material—Mixed Polish Herbs, INCT-MPH-2. The marked value obtained as the average of 4 samples for Cd was: 0.21 ± 0.004 μg/g, (certified value of 0.199 ± 0.015 μgCd/g, 95% recovery), for Pb: 2.12 ± 0.004 μg/g, (certified value of 2.16 ± 0.23 μgPb/g, 98% recovery). For Hg, the average concentration for 3 repetitions was 0.01919 ± 0.004 μgHg/g, and the certified value was 0.018 ± 0.002. The recovery value was 107%.

### 2.4. Statistical Analysis

The statistical analysis of results of the markings was made using Statistica (12, StatSoft, Cracow, Poland). The use of non-parametric analyses depended on the distribution of concentrations of the tested elements. Statistical conclusions were based on median values and non-parametric tests were used to assess the importance of differences between groups of results (U Mann-Whitney test for two samples, ANOVA rang Kruskal-Wallis test for multiple samples), significance level of *p* < 0.05 was assumed to be statistically significant.

## 3. Results

The concentration of Pb, Cd and Hg in the examined plant samples is shown in Table 1. Among the heavy metals analysed, the highest concentration was determined for Pb (4.67–23.8 mg/kg) and for Cd (0.01–1.51 mg/kg), and the lowest for Hg (0.005–0.028 mg/kg). The variability of content determined by the value of the coefficient of variation was the highest for Cd, then for Hg. The lowest degree of variation in the tested plant samples was observed in the concentration of Pb. The level of concentration of heavy metals was determined for 6 species of herbs—Table 1. The average concentration of all tested metals (Pb, Cd and Hg) was the lowest in St. John’s wort. Other plant species showed variable concentrations of heavy metals. Pb concentration was the highest in nettle, Cd—in yarrow, and Hg—in horsetail—Table 1.

**Figure 2:**
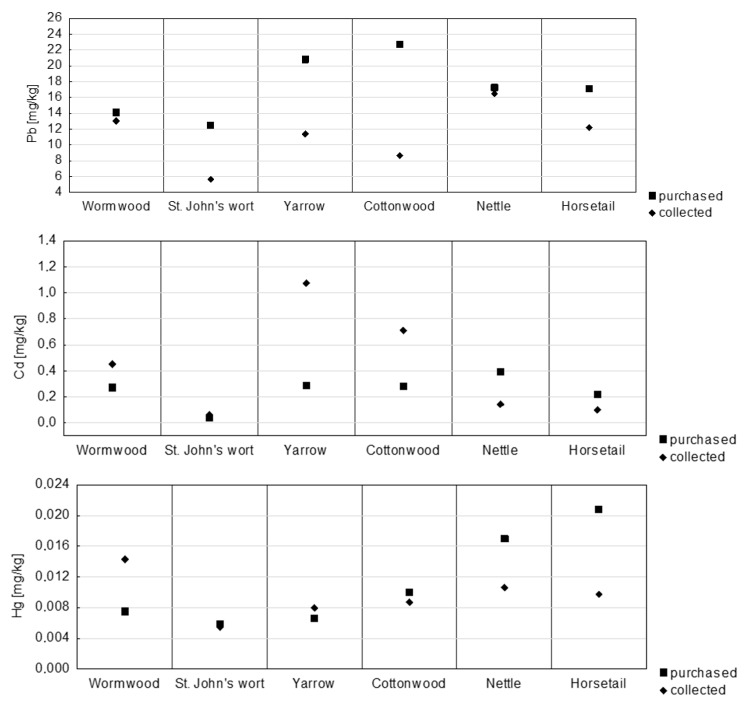
Comparison of concentration of Pb, Cd and Hg in self-collected and purchased plants (mg/kg).

Differences in the concentration of Pb, Cd and Hg in samples of herbs originating from purchased products and prepared from wild plants were analysed in the study—Figure 2.

The average concentration of Pb in plant samples determined for commercial products was statistically significantly higher than in wild plant samples (*p* = 0.0014, U Mann-Whitney test). The higher concentration of Pb in samples of purchased plants was indicated for all plant species tested. For example, in commercial samples of cottonwood, the concentration of Pb was more than twice as high as in samples of wild plants (respectively: 22.8 mg/kg Pb, 8.65 mg/kg Pb)—Figure 2. Similarly, in the case of nettle and horsetail the purchased samples had a higher concentration of Cd and Hg than the samples prepared from wild herbs. The situation was different for wormwood and yarrow—the samples prepared from wild herbs had higher average concentrations of Cd and Hg compared to the purchased samples—Figure 2. There were no statistically significant differences in the concentrations of Cd and Hg between purchased and self-collected plants.

**Figure 3:**
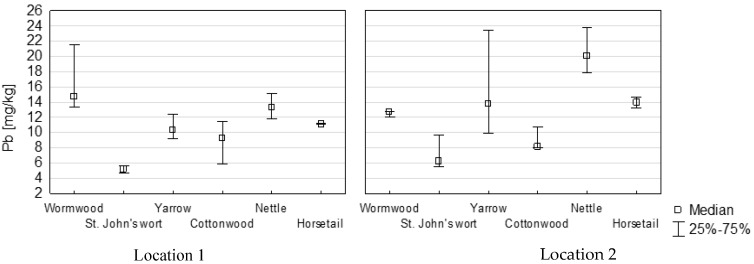

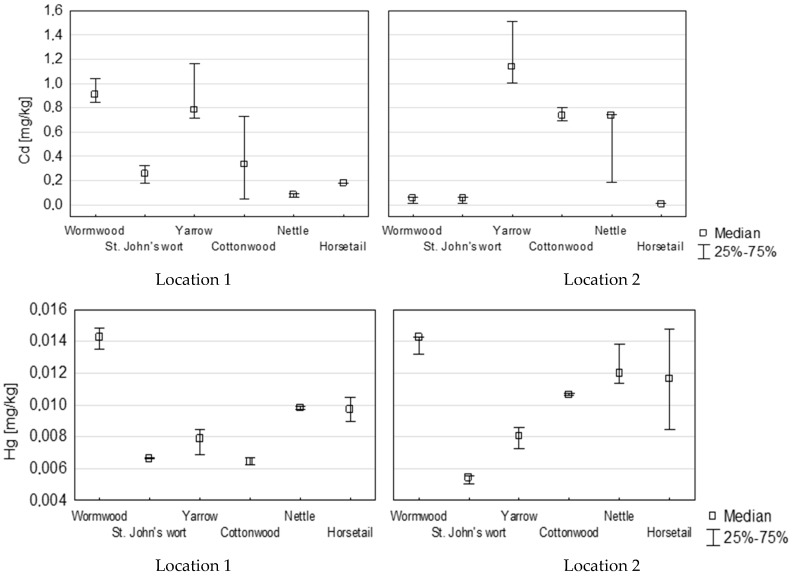
Concentration of Pb, Cd and Hg in self-collected species of herbs (mg/kg).

The study analysed the effect of the plant’s growing location on the concentration of the studied heavy metals in herbs. Higher concentrations of Pb and Hg were recorded for all plant samples self-collected at Location 2 (Leboszowice). Differently, the concentration of Cd was higher in plant samples from Location 1 (Niedośpielin). Among the studied plant species self-collected, the highest concentration of Cd was in wormwood and yarrow, and the smallest in nettle—Figure 3.

## 4. Discussion

In the light of the World Health Organisation’s (WHO) data, the percentage of people using medicinal plants is 70–80% [29]. Herbal raw materials (leaves, herbs, rhizomes, roots, oils) can be sources of undesirable toxic components, including heavy metals. The maximum values for heavy metals in medicinal plants oral intake determined by the WHO are as follows: less than 10 mgPb/kg and 0.3 mgCd/kg [29].

The concentration of toxic metals in cosmetic plants and plants used in cosmetology was widely discussed [5,14,18,20,30,31,32,33,34,35,36,37,38]. In Brazil, the standard quality control of these products is not always enforced, and their quality, efficacy and safety is unclear. The results show the need for a systematic control of toxic heavy metals in plants used as medicines and cosmetics [36]. The personal care products like soap, body and hair creams can expose users to significant levels of heavy metals [38]. In the study by Adepoju-Bello et al. [5], arsenic, cadmium, lead, mercury and nickel were determined in various brand of creams, lipsticks and lip-glosses. From the results, the toxic metals were present in low quantities. The continuous use of cosmetic products contaminated with such heavy metals may however cause slow release of these metals into the human body and cause harmful effects to the consumers over time. Extensive use of such products should be avoided [5]. The symptoms associated with mercury poisoning among persons who used a Mexican beauty cream containing mercurous chloride have been reported in humans living in Texas near the Mexico border [18]. Some cases of poisonings owing to the use of skin creams containing mercury have been reported in the past, Hg-containing skin-lightening creams are still commonly used in many developing countries [20].

In the author’s research, the concentrations of Pb, Cd and Hg were assessed in plants with potential cosmetic applications. The analysis of co-occurrence of the studied metals showed a positive correlation between the concentration of Pb and Hg (*r* = 0.44). For all plants self-collected in the natural environment, the quantitative proportions between Pb and Hg were determined by the statistically significant correlation coefficient (0.63), which may indicate a common source of pollution with these elements. The natural environment in Poland is characterized by a significant degree of pollution, which translates into the concentration of heavy metals in various elements, also in plants [1,39,40,41,42]. The main source of environmental pollution is industry. It affects the quality of the air, but also water and soil. Depending on what kinds of soils and in which geographical area a particular species of medicinal plant grows, raw materials obtained from such plants will be polluted with different heavy metals [43,44]. In nettle collected by Tokalioglu [45], the average concentration of Pb was 1.59 mg/kg, and in St. John’s wort 1.17 mg/kg. These values were much lower than those indicated by our research (respectively: 11.8 mg/kg Pb, 6.23 mg/kg Cd). Also in Turkey, the concentration of the tested elements in medicinal plants was lower [46]. In samples of wild plants in Poland, the concentration of heavy metals is higher [47]. Area diversity is also observed. The average concentration of Pb in nettle growing in the studied area (16.5 mg/kg) (Figure 2) was higher than in nettle collected in the Beskidy Mountains (Poland) determined by Baranowska et al. [47], amounting to 7.23 mg/kg and lower than for plants growing near an express way (51 mg/kg). The highest concentration of Pb was recorded in nettle samples from Location 2 (20.2 mg/kg). The region in the area of which there is Location 2 is characterised by a very high degree of industrialization in Poland [48]. The concentration of Hg in soils of this region (Upper Silesia Region) is one of the highest in the country and can reach 5130 μgHg/kg [42]. The concentration of Hg in the tested medicinal plants self-collected in this area was significantly higher in 3 out of 6 tested species (cottonwood, nettle and horsetail). St. John’s wort, wormwood, and yarrow had a comparable concentration of Hg regardless of where the plants were harvested. The distance between the plant collection locations was approximately 160 km. However, the concentration of heavy metals determined in the samples indicated heterogeneity of the environment of the studied areas. For example, in nettle growing in Location 1, the level of Pb concentration (13.3 mg/kg) was lower than in Location 2 (20.1 mg/kg). In nettle samples self-collected in this location, lower concentrations of Hg and Cd were observed as well. The average concentration of Cd in the nettle tested (0.24 mg/kg) was the result of concentrations of this metal in nettle from a mountain area (0.27 mg/kg) and from a motorway area (1.42 mg/kg) obtained by Baranowska et al. [47]. Tokalioglu’s research [45] showed a higher concentration of Cd in nettle than in St. John’s wort. Similarly, nettle samples examined in this research had a higher concentration of Pb than St. John’s wort samples. In the case of plants self-collected in Location 2, the average concentration of Pb was almost 3 times higher in nettle samples compared to St. John’s wort—Figure 3.

Unlike the research by Gasser et al. [49], Chizzola [50] and Baranowska et al. [47]. Individual plant species show differentiated susceptibility as a response to toxic agents, including heavy metals [51,52,53]. The content of metals in plants is the result of a variety of factors, including environmental, and the specificity of a given species. Moreover, the plant collection period can affect the concentration of elements.

The results of studies by Sembratowicz et al. [40] show that nettle collected during two growing seasons shows a higher concentration of heavy metals in the summer months (July–August) rather than in the spring months (April–May), depending on the season. This is due to the apparent dilution of metals contained in the plant during its intense growth in the spring period [40].

In Romania, Badea studied the concentration of metals in medicinal plants [54]. The research concerned the same elements, and the concentration of Pb in St. John’s wort was 7.21 mg/kg, Cd was 0.12 mg/kg, and Hg was 0.005 mg/kg [54]. These results were very similar to those in our research (8.1 mg/kg Pb, 0.102 mg/kg Cd, 0.006 mg/kg Hg). Differences in concentrations of metals between the results obtained and Badea’s results [54] occurred in case of wormwood. Wormwood collected in Romania had a lower concentration of Pb as compared to own results (5.37–14.42 mg/kg), Cd (0.118–0.434 mg/kg) and Hg (0.005–0.012 mg/kg).

In the research conducted, the concentration of Cd, Pb and Hg in self-collected plant samples and commercial phytotherapy products was compared. The co-occurrence of metals between the commercial herbal products tested did not show any statistical significance. In addition, the concentration of Cd and Pb had a negative correlation coefficient. This indicates that changes in the concentration of heavy metals in herbal products are not subject to significant interactions and may have different origins. Also, the concentration of metals in other commercially available herbs varies [39]. The average concentration of Cd in various commercial formulas was 0.16–0.68 mg/kg [39], which was in line with the results of our research (the average concentration of Cd in herbal products we tested was 0.28 mg/kg). A lower concentration of Cd in herbal nettle products purchased in Poland was indicated by Fijałek et al. [41] (0.057–0.101 mg/kg Cd). In general, commercial products containing medicinal herbs had lower concentrations of Cd and Hg than samples prepared from self-collected wild plants. This applies especially to the selected plant species—St. John’s wort and yarrow. In the case of the concentration of Pb, the results of the research indicated that the samples of herbal material tested had a higher concentration of Pb than the samples of wild plants analysed. The differences between the concentration of Pb in plant samples self-collected independently and purchased in stores were statistically significant (*p* = 0.004). The statistically significantly higher concentration of Pb in commercial products was indicated for all tested plant species. The greatest differences in the concentration of Pb were found in cottonwood (8.65 mg/kg in naturally growing samples and 22.8 mg/kg in commercial samples). A comparable concentration of Pb was found only for nettle—Figure 2. The statistically higher concentration of Pb in samples prepared from commercial formulations compared to self-collected plant samples and the lack of statistically significant correlations between the tested metals may suggest contamination with this metal during technological plant processing. Prior to introducing the products onto the market, the plants are subjected to a multi-stage technological process. Further stages of production (storage, cleaning, drying, grinding) can cause contamination of the harvested plant material.

The maximum values for heavy metals in medicinal plants oral intake are as follows: less than 10 mg Pb/kg and 0.3 mgCd/kg [55]. The average concentration in the tested plant samples exceeds the acceptable levels (13.6 mgPb/kg and 0.41 mgCd/kg). However, it should be remembered that the standards have been set for medicinal plants that are mainly consumed orally. Whereas plants used for cosmetic purposes are mainly used externally.

According to Pharmeuropa, in the monograph entitled “Herbal Drugs” [56] acceptable limits are as follows: Pb 5 mg/kg, Cd 0.5 mg/kg and Hg 0.1 mg/kg for oral intake. In this case, the concentration of Cd and Hg in the tested plants is below, and Pb exceeds this value by more than 100%.

According to Canadian regulations, polluting cosmetics with metals should be reduced as much as technically feasible. Comparing data collected with limits of pollutants acceptable in cosmetology in Canada, a permissible value of 10 mg/kg exceeds the concentration of lead, in case of the cadmium of 3 mg/kg and Hg indicated concentrations are 1 mg/kg below these values [57].

In the United States cosmetics are subject to the Federal Food, Drug and Cosmetics Act—FD&C Act. This law prohibits the use of ingredients that are toxic or have a harmful effect in conditions of use. This is supervised by the Food and Drug Administration (FDA). For mercury the limit is 1 mg/kg, whereas for mercury in eye make-up products it cannot exceed 65 mg/kg, these values are not exceeded in the tested plants [58].

## 5. Conclusions

The species of plants tested (nettle, yarrow, St. John’s wort, wormwood, horsetail, cottonwood), with potential uses in cosmetics and cosmetology, have different concentrations of metals depending on the plant species tested and the area of collection.

The highest concentration of Pb was found in nettle, Cd in yarrow, and Hg in horsetail.

The average concentration of all metals studied was the lowest in St. John’s wort (8.2 mg/kg), and the highest in nettle (17.4 mg/kg).

A statistically significant higher concentration of Pb was found in samples of herbal products, compared to naturally self-collected plants. Purchased herbal products can be a greater source of exposure (in this case to Pb) than self-collected and prepared ones. 

The use of medicinal plants for cosmetic purposes relates mainly to external application. Despite the little importance of this intoxication path, it should be remembered that heavy metals may accumulate in the body and lead to health issues.

## Figures and Tables

**Table 1:** Concentration of Pb, Cd and Hg in plants (wormwood, St. John’s wort, yarrow, cottonwood, nettle, horsetail) (mg/kg).

Herbal Plants	Metal	Mean	Median	Min	Max	SD	CV%
All plants	Pb	13.61	12.71	4.673	23.76	5.318	39.1
	Cd	0.412	0.300	0.010	1.511	0.396	96.3
	Hg	0.010	0.010	0.005	0.028	0.004	44.0
Wormwood	Pb	14.42	13.03	8.545	21.60	4.276	29.7
	Cd	0.434	0.270	0.012	1.040	0.429	99.0
	Hg	0.012	0.014	0.007	0.015	0.003	24.6
St. John’s wort	Pb	8.106	6.229	4.673	14.83	3.632	44.8
	Cd	0.102	0.059	0.010	0.325	0.113	110
	Hg	0.006	0.006	0.005	0.007	0.001	10.3
Yarrow	Pb	15.07	13.12	9.171	23.44	5.845	38.8
	Cd	0.864	0.896	0.013	1.511	0.455	52.6
	Hg	0.008	0.008	0.007	0.009	0.001	10.7
Cottonwood	Pb	12.38	9.970	5.860	23.67	6.673	53.9
	Cd	0.489	0.515	0.046	0.804	0.284	58.0
	Hg	0.009	0.010	0.006	0.011	0.002	23.2
Nettle	Pb	17.05	16.57	11.81	23.76	3.922	23.0
	Cd	0.336	0.244	0.065	0.745	0.285	84.9
	Hg	0.013	0.011	0.010	0.022	0.004	33.6
Horsetail	Pb	14.07	13.96	11.15	17.32	2.698	19.2
	Cd	0.139	0.117	0.010	0.394	0.148	107
	Hg	0.014	0.012	0.008	0.028	0.007	53.2

## References

[1] Szynkowska M.I., Pawlaczyk A., Leśniewska E., Paryjczak T. (2009). Toxic metal distribution in rural and urban soil samples affected by industry and traffic. Pol. J. Environ. Stud..

[2] Pivic R., Stanojkovic-Sebic A., Josic D., Dinic Z. (2014). Evaluation of the heavy metals content in soil and plant material at different distances from the from the motorway e75 in the section Belgrade-Presevo (Serbia). Bulg. J. Agric. Sci..

[3] Siti Zulaikha R., Sharifah Norkhadijah S.I., Praveena S.M. (2015). Hazardous ingredients in cosmetics and personal care products and health concern: A review. Public Health Res..

[4] Łopaciuk A., Łoboda M. Global beauty industry trends in the 21st century. Proceedings of the Management, Knowledge and Learning International Conference.

[5] Adepoju-Bello A.A., Oguntibeju O.O., Adebisi R.A., Okpala N., Coker H.A.B. (2012). Evaluation of the concentration of toxic metals in cosmetic products in Nigeria. Afr. J. Biotechnol..

[6] Nordberg G.F., Fowler B.A., Nordberg M., Friberg L. (2007). Handbook on the Toxicology of Metals.

[7] Ayenimo J.G., Yusuf A.M., Adekunle A.S., Makinde O.W. (2010). Heavy metal exposure from personal care products. Bull. Environ. Contam. Toxic..

[8] Al-Dayel O., Hefne J., Al-Ajyan T. (2011). Human exposure to heavy metals from cosmetics. Orient. J. Chem..

[9] Nouioui M.A., Mahjoubi S., Ghorbel A., Yahia M.B.H., Amira D., Ghorbel H., Hedhili A. (2016). Health risk assessment of heavy metals in traditional cosmetics sold in Tunisian local markets. Int. Sch. Res..

[10] Volpe M.G., Nazzaro M., Coppola R., Rapuano F., Aquino R.P. (2012). Determination and assessments of selected heavy metals in eye shadow cosmetics from China, Italy, and USA. Microchem. J..

[11] Mayildurai R., Ramasubbu A., Velmani N. (2015). ICP—OES investigations of heavy metal contents in cosmetic products. J. Pharm. Res..

[12] Al Aboud A.M. (2011). Plants and dermatology: A panoramic view. J. Pak. Assoc. Dermatol..

[13] Al-Saleh I., Al-Enazi S., Shinwari N. (2009). Assessment of lead in cosmetic products. Regul. Toxicol. Pharmacol..

[14] Peregrino C.P., Moreno M.V., Miranda S.V., Rubio A.D., Leal L.O. (2011). Mercury levels in locally manufactured Mexican skin-lightening creams. Int. J. Environ. Res. Public Health.

[15] Zhou H., Yang W.T., Zhou X., Liu L., Gu J.F., Wang W.L., Zou J.L., Tian T., Peng P.Q., Liao B.H. (2016). Accumulation of heavy metals in vegetable species planted in contaminated soils and the health risk assessment. Int. J. Environ. Res. Public Health.

[16] Wu Q., Li W.K., Zhou Z.P., Li Y.Y., Xiong T.W., Du Y.Z., Wei L.X., Liu J. (2016). The Tibetan medicine Zuotai differs from HgCl_2_ and MeHg in producing liver injury in mice. Regul. Toxicol. Pharmacol..

[17] Benz M.R., Lee S.H., Kellner L., Döhlemann C., Berweck S. (2011). Hyperintense lesions in brain MRI after exposure to a mercuric chloride-containing skin whitening cream. Eur. J. Pediatr..

[18] Weldon M.M., Smolinski M.S., Maroufi A., Hasty B.W., Gilliss D.L., Boulanger L.L., Balluz L.S., Dutton R.J. (2000). Mercury poisoning associated with a Mexican beauty cream. West. J. Med..

[19] McKelvey W., Jeffery N., Clark N., Kass D., Parsons P.J. (2011). Population-based inorganic mercury biomonitoring and the identification of skin care products as a source of exposure in New York City. Environ. Health Perspect..

[20] Borowska S., Brzóska M.M. (2015). Metals in cosmetics: Implications for human health. J. Appl. Toxicol..

[21] Skotnicka-Klonowicz G., Rutkowska A., Janota A., Lewartowska-Nyga D., Śmigielski J., Grochocińska P. (2014). Acute accidental and intentional poisonings in children and adolescents in material of Clinical Emergency Department for Children of University Hospital No 4 in Łódź. Probl. Hig. Epidemiol..

[22] Aburjai T., Natsheh F.M. (2003). Plants used in cosmetic. Phytother. Res..

[23] Kiełtyka-Dadasiewicz A. (2016). Rośliny w Nowoczesnej Kosmetologii. Wydawnictwo Akademickie Wyższej Szkoły Społeczno-Przyrodniczej im.

[24] Campos M.M., Tonuci H., Silva S.M., de S Altoé B., de Carvalho D., Kronka E.A., Pereira A.M., Bertoni B.W., de C França S., Miranda C.E. (2009). Determination of lead content in medicinal plants by pre-concentration flow injection analysis-flame atomic absorption spectrometry. Phytochem. Anal..

[25] Dasgupta A., Hammett-Stabler C.A. (2011). Herbal Supplements: Efficacy, Toxicity, Interactions with Western Drugs, and Effects on Clinical Laboratory Tests.

[26] Sumit K., Vivek S., Sujata S., Ashish B. (2012). Herbal cosmetics: Used for skin and hair. Inven. J..

[27] Pasieczna A. (2012). Rtęć w glebach obszarów zurbanizowanych. Prz. Geol..

[28] Berkowska E., Kijak R. (1992). Ograniczenie Emisji Zanieczyszczeń z Pojazdów Mechanicznych w Polsce. Benzyny Niskoołowiowe i Bezołowiowe.

[29] World Health Organization (WHO) (2002). Tradiciolan Medicine Strategy 2002–2005.

[30] Saper R.B., Kales S.N., Paquin J., Burns M.J., Eisenberg D.M., Roger M.D., Davis B., Phillips R.S. (2004). Heavy metal content of ayurvedic herbal medicine. J. Am. Med. Assoc..

[31] Ernst E. (2002). Toxic heavy metals and undeclared drugs in Asian herbal medicines. Trends Pharmacol. Sci..

[32] Chan K. (2003). Some aspects of toxic contaminants in herbal medicines. Chemosphere.

[33] Sipahi H., Charehsaz M., Sonmez I., Soykut B., Erdem O., Aydin A. (2014). Assessment of cadmium, lead, and nickel levels in hair care products marketed in Turkey. J. Cosmet. Sci..

[34] Ozbek N., Akman S. (2016). Determination of lead, cadmium and nickel in hennas and other hair dyes sold in Turkey. Regul. Toxicol. Pharmacol..

[35] Gomez M.R., Cerutti S., Sombra L.L., Silva M.F., Martínez L.D. (2007). Determination of heavy metals for the quality control in argentinian herbal medicines by ETAAS and ICP-OES. Food Chem. Toxicol..

[36] Caldasa E.D., Machado L.L. (2004). Cadmium, mercury and lead in medicinal herbs in Brazil. Food Chem. Toxicol..

[37] Orisakwe O.E., Okolo K.O., Igweze Z.N., Ajaezi G.C., Udowelle N.A. (2016). Potential hazards of toxic metals found in toothpastes commonly used in Nigeria. Rocz. Państw. Zakł. Hig..

[38] Ayenimo J.G., Yusuf A.M., Doherty W.O., Ogunkunle O.A. (2010). Iron, lead, and nickel in selected consumer products in Nigeria: A potential public health concern. Toxicol. Environ. Chem..

[39] Klany P., Fijałek Z., Daszczuk A., Ostapczuk P. (2007). Determination of selected microelements in polish harbs and their infusion. Sci. Total Environ..

[40] Sembratowicz I., Rusinek E., Ognik K., Truchliński J. (2009). Concentrations of trace elements and hevy metals at selected medicinal plants haversted in two vegetation periods. Herba Pol..

[41] Fijałek Z., Sołtyk K., Łozak A., Kominek A., Ostapczuk P. (2003). Determination of some micro- and macroelements in preparations made from peppermint and nettle *Urtica dioica* leaves. Pharmazie.

[42] Gworek B., Dmuchowski W., Gozdowski D., Koda E., Osiecka R., Borzyszkowski J. (2015). Influence of a municipal waste landfill on the spatial distribution of mercury in the enviroment. PLoS ONE.

[43] Annan K., Dickson R.A., Amponsah I.K., Nooni I.K. (2013). The hevy metal of some selected medicinal plants sampled from different geographical locations. Pharmacogn. Res..

[44] Obiora G.U., Chibuike S.C. (2014). Heavy metal polluted soils: Effect on plants and bioremediation methods. Appl. Environ. Soil Sci..

[45] Tokalioglu S. (2012). Determination of trace elements in commonly consumed medicinal herbs by ICP-MS and multivariate analysis. Food Chem..

[46] Basgel S., Erdemoglu S.A. (2006). Determination of mineral and trace elements in some medicinal herbs and their infusions consumed in Turkey. Sci. Total Environ..

[47] Baranowska I., Srogi K., Włochowicz A., Szczepanik K. (2002). Determination of heavy metal contents in samples of medicinal herbs. Pol. J. Environ. Stud..

[48] Bell J.N.B., Treshow M. (2002). Zanieczyszczenie Powietrza a Życie Roślin.

[49] Gasser U., Klier B., Kuhn A.V., Steinhoff B. (2009). Current findings on the heavy metal content in harbal drugs. Pharmeur. Sci. Notes.

[50] Chizzola R. (2012). Metallic mineral elements and heavy metals in medicinal plants. Med. Aromat. Plant Sci. Biotechnol..

[51] Memon A.R., Schröder P. (2009). Implications of metal accumulation mechanisms to phytoremediation. Environ. Sci. Pollut. Res. Int..

[52] Jin C.W., Zheng S.J., He Y.F., Zhou G.D., Zhou Z.X. (2005). Lead contamination in tea garden soils and factors affecting its bioavailability. Chemosphere.

[53] Rozkrut D. (2016). Statistical Yearbook of Industry—Poland.

[54] Badea D.N. (2015). Determination of potentially toxic heavy metals (Pb, Hg, Cd) in popular medicinal herbs in the coal power plant area. Rev. Chim..

[55] World Health Organization (WHO) (1999). Monographs on Selected Medicinal Plants.

[56] Drugs H. (2008). Monograph 1433. Pharmeuropa.

[57] Government of Canada (2012). Guidance on Heavy Metal Impurities in Cosmetics.

[58] US Food and Drug Administration (2017). Federal Food, Drug and Cosmetic Act (FD&C Act). https://www.fda.gov/RegulatoryInformation/LawsEnforcedbyFDA/FederalFoodDrugandCosmeticActFDCAct/default.htm.

